# Genetic Improvement for Resistance to Black Sigatoka in Bananas: A Systematic Review

**DOI:** 10.3389/fpls.2021.657916

**Published:** 2021-04-21

**Authors:** Julianna M. S. Soares, Anelita J. Rocha, Fernanda S. Nascimento, Adriadna S. Santos, Robert N. G. Miller, Cláudia F. Ferreira, Fernando Haddad, Vanusia B. O. Amorim, Edson P. Amorim

**Affiliations:** ^1^Department of Biological Sciences, Feira de Santana State University, Feira de Santana, Brazil; ^2^Secretariat of Education of the State of Bahia, Salvador, Brazil; ^3^Department of Cell Biology, University of Brasília, Brasília, Brazil; ^4^Embrapa Mandioca e Fruticultura, Cruz das Almas, Brazil

**Keywords:** black Sigatoka, *Musa* spp., *Pseudocercospora fijiensis*, genetic resistance, state-of-the-art

## Abstract

Bananas are an important staple food crop in tropical and subtropical regions in Asia, sub-Saharan Africa, and Central and South America. The plant is affected by numerous diseases, with the fungal leaf disease black Sigatoka, caused by *Mycosphaerella fijiensis* Morelet [anamorph: *Pseudocercospora fijiensis* (Morelet) Deighton], considered one of the most economically important phytosanitary problem. Although the development of resistant cultivars is recognized as most effective method for long term control of the disease, the majority of today's cultivars are susceptible. In order to gain insights into this pathosystem, this first systematic literature review on the topic is presented. Utilizing six databases (PubMed Central, Web of Science, Google Academic, Springer, CAPES and Scopus Journals) searches were performed using pre-established inclusion and exclusion criteria. From a total of 3,070 published studies examined, 24 were relevant with regard to the *Musa-P. fijiensis* pathosystem. Relevant papers highlighted that resistant and susceptible cultivars clearly respond differently to infection by this pathogen. *M. acuminata* wild diploids such as Calcutta 4 and other diploid cultivars can harbor sources of resistance genes, serving as parentals for the generation of improved diploids and subsequent gene introgression in new cultivars. From the sequenced reference genome of *Musa acuminata*, although the function of many genes in the genome still require validation, on the basis of transcriptome, proteome and biochemical data, numerous candidate genes and molecules have been identified for further evaluation through genetic transformation and gene editing approaches. Genes identified in the resistance response have included those associated with jasmonic acid and ethylene signaling, transcription factors, phenylpropanoid pathways, antioxidants and pathogenesis-related proteins. Papers in this study also revealed gene-derived markers in *Musa* applicable for downstream application in marker assisted selection. The information gathered in this review furthers understanding of the immune response in *Musa* to the pathogen *P. fijiensis* and is relevant for genetic improvement programs for bananas and plantains for control of black Sigatoka.

## Introduction

Bananas and plantains (*Musa* spp.) are important commodity fruit crops in terms of trade and consumption, and represent the fourth most important staple food worldwide (Weber et al., 2017). World production in 2018 was ~154.5 million tons, of which 74% were bananas and 26% plantains, grown over a total area of 11.3 million hectares (FAOSTAT, 2021).

Although bananas originated in Southwest Asia and the Western Pacific region, popularity and economic importance occurred following introduction to Africa, Latin and Central America and the South Pacific (Valmayor, 2001; De Langhe et al., 2009). The vast majority of banana and plantain cultivars originated from hybrids of the two wild diploid species, *Musa acuminata Colla* (genome A) and *M. balbisiana Colla* (genome B). Such crossings resulted in a series of diploids, triploids and tetraploids, with genomic groups classified as AA, AB, AAA, AAB, ABB, AABB, AAAB, and ABBB (Simmonds and Shepherd, 1955).

Banana and plantain production are affected by various pests and diseases, including bacterial wilt (Addy et al., 2016), nematodes (Seenivasan, 2017), Fusarium wilt (Dita et al., 2018; Arinaitwe et al., 2019) and yellow and black Sigatoka diseases (Ferreira et al., 2004; Timm et al., 2016). Black Sigatoka, caused by the fungus *Mycosphaerella fijiensis* Morelet [anamorph: *Pseudocercospora fijiensis* (Morelet) Deighton], can result in considerable negative economic impact, affecting both bananas and plantains across all global growing regions. Whilst chemical control is considered efficient, problems can arise from indiscriminate use, where this approach is detrimental to human health and the environment. Agrochemical-based control is also expensive (Churchill, 2011), with data indicating ~US$ 1,000/ha spent on disease control annually in large plantations, corresponding to up to 30% of the total production costs (Churchill, 2011; Alakonya et al., 2018). Another important factor to be considered with dependency on agrochemicals is the possible medium- and long-term selection for pathogen strains acquiring resistance to fungicides, potentially reducing effectiveness (Churchill, 2011; Chong, 2016; Friesen, 2016; Rodríguez-García et al., 2016; Oiram-Filho et al., 2019).

Rain splash of asexual conidia and airborne dispersal of sexual ascospores enable effective spread of *P. fijiensis* (Churchill, 2011; Rodríguez-García et al., 2016; Alakonya et al., 2018). The onset of the first symptoms of the disease typically occurs between 7 and 14 days after contamination, depending on local environmental conditions. Following fungal penetration of leaf stomata, colonization of intercellular spaces and subsequent necrotic damage then decrease the photosynthetic capacity of the plant, reducing the quantity and quality of fruits (Churchill, 2011; Alakonya et al., 2018; Cruz-Martín et al., 2018).

Whilst increased understanding of the genetic structure of pathogen populations and their evolution are important components to consider in strategies for *Musa* genetic improvement and management of the disease (Churchill, 2011), the identification at the molecular level of host genes related to resistance to *P. fijiensis* will advance improvement of banana through both assisted selection and genetic engineering (Mendoza-Rodríguez, 2014). Our understanding of the innate immune system in plants has advanced considerably in recent years, with challenge by pathogen molecules known to activate host receptor proteins for pathogen recognition. In a first layer of the immune response, referred to as pathogen-associated molecular pattern (PAMP)-triggered immunity (PTI), or non-host resistance, host cell surface pattern recognition receptors (PRRs) (Dangl and Jones, 2001; Monaghan and Zipfel, 2012) recognize conserved pathogen-associated molecular patterns (PAMPs) (Jones and Dangl, 2006; Boutrot and Zipfel, 2017) such as bacterial flagellin and fungal cell wall chitin (Felix et al., 1999; Wan et al., 2004; Thomma et al., 2011; Zipfel, 2014; Gong et al., 2020). Plant PRRs, which include receptor-like kinases (RLKs) and receptor-like proteins (RLPs), generally contain extracellular domains with a capacity for ligand binding, transmembrane domains and intracellular domains (Zipfel, 2014). Activation of PRRs following PAMP recognition will trigger intracellular signaling and plant defense responses to block pathogen advance in the host. These include reactive oxygen species (ROS), mitogen-activated protein kinase (MAPK) cascades and Ca^2+^ signaling influx (Chisholm et al., 2006; Dangl et al., 2013; Li et al., 2016). Race-specific pathogen effector proteins, or avirulence (Avr) proteins, when secreted into the host cell by evolving pathogens, by contrast, can suppress PTI and result in an effector-triggered susceptibility (ETS) with subsequent disease (Jones and Dangl, 2006; Boller and Felix, 2009). In a second layer of the plant immunity defense response, intracellular nucleotide-binding and leucine-rich repeat domain intracellular resistance receptors (NLRs) recognize directly or indirectly evolved pathogen effectors, activating effector-triggered Immunity (ETI) (Jones and Dangl, 2006). As a more intense response, this again involves calcium ion signaling and ROS, together with transcriptional reprogramming, changes in levels of plant hormones salicylic acid (SA) and jasmonic acid (JA) (Creelman and Mullet, 1995), and the accumulation of pathogenesis-related (PR) proteins (Gururani et al., 2012). Such a suite of responses can also involve the signature hypersensitive response, comprising a programmed and localized host cell death at the site of infection (Jones and Dangl, 2006; Coll et al., 2011; Cui et al., 2015), effectively limiting pathogen advance. Subsequent systemic acquired resistance (SAR) can also occur, conferring a broad spectrum response in the host that heightens resistance to any subsequent pathogen attack (Dong, 2001; Spoel and Dong, 2012).

The pathosystem *Musa* spp. x *P. fijiensis* is complex, given the characteristics of the polyploid host and the morphophysiology of the hemibiotrophic fungus. To date, there have been few studies on the biology of this hemibiotroph and the mode of action of genes involved in the host-pathogen interaction (Cavalcante et al., 2011; Torres et al., 2012; Mendoza-Rodríguez, 2014; Arango-Isaza et al., 2016). Similarly, although the genus *Musa* has been relatively widely studied with regard to molecular marker development and analysis of genetic diversity, with whole genome sequences also developed in recent years for *M. acuminata* and related species, detailed investigation and validation of gene function in immune responses in different *Musa*-pathogen interactions remains limited (Sun et al., 2009; Li et al., 2012; Wang et al., 2012; Bai et al., 2013; Castañeda et al., 2017). With regard to *Musa-Pseudocercospora* interactions, candidate gene discovery has broadly been undertaken through analysis of gene analogs and through transcriptomics approaches (Miller et al., 2008, 2011; Emediato et al., 2009, 2013; Portal et al., 2011; D' Hont et al., 2012; Passos et al., 2012, 2013; Sulliman et al., 2012; Timm et al., 2016).

Systematic literature reviews are analyses that gather and critically evaluate compiled data from previously published scientific investigations. Such an approach for synthesis of findings is widely employed in medical fields, enabling, in a single document, relevant information to be gathered on a specific topic, for example on a disease or active ingredient in medicines and potential side effects (Falcomer et al., 2019; Jones et al., 2020). For *Musa spp*., there have only been two studies using such a strategy, with focus on plant physiology associated with water deficit and on fruit consumption preferences (Santos et al., 2018; Falcomer et al., 2019).

Accumulation of knowledge on host genetics and genomics, resistance and defense mechanisms, together with information on methods and tools employed in development of resistance to black Sigatoka, is relevant for genetic improvement strategies for development of resistant cultivars. This systematic review synthesizes relevant literature published in the last 10 years on genetic improvement of banana with a focus on black Sigatoka, to answer the following question: what are the strategies adopted in genetic improvement that aim to reduce the impact of black Sigatoka on banana plants? To our knowledge, this is the first systematic review applied to the *Musa* spp. x *P. fijiensis* pathosystem.

## Materials and Methods

**Figure 1:**
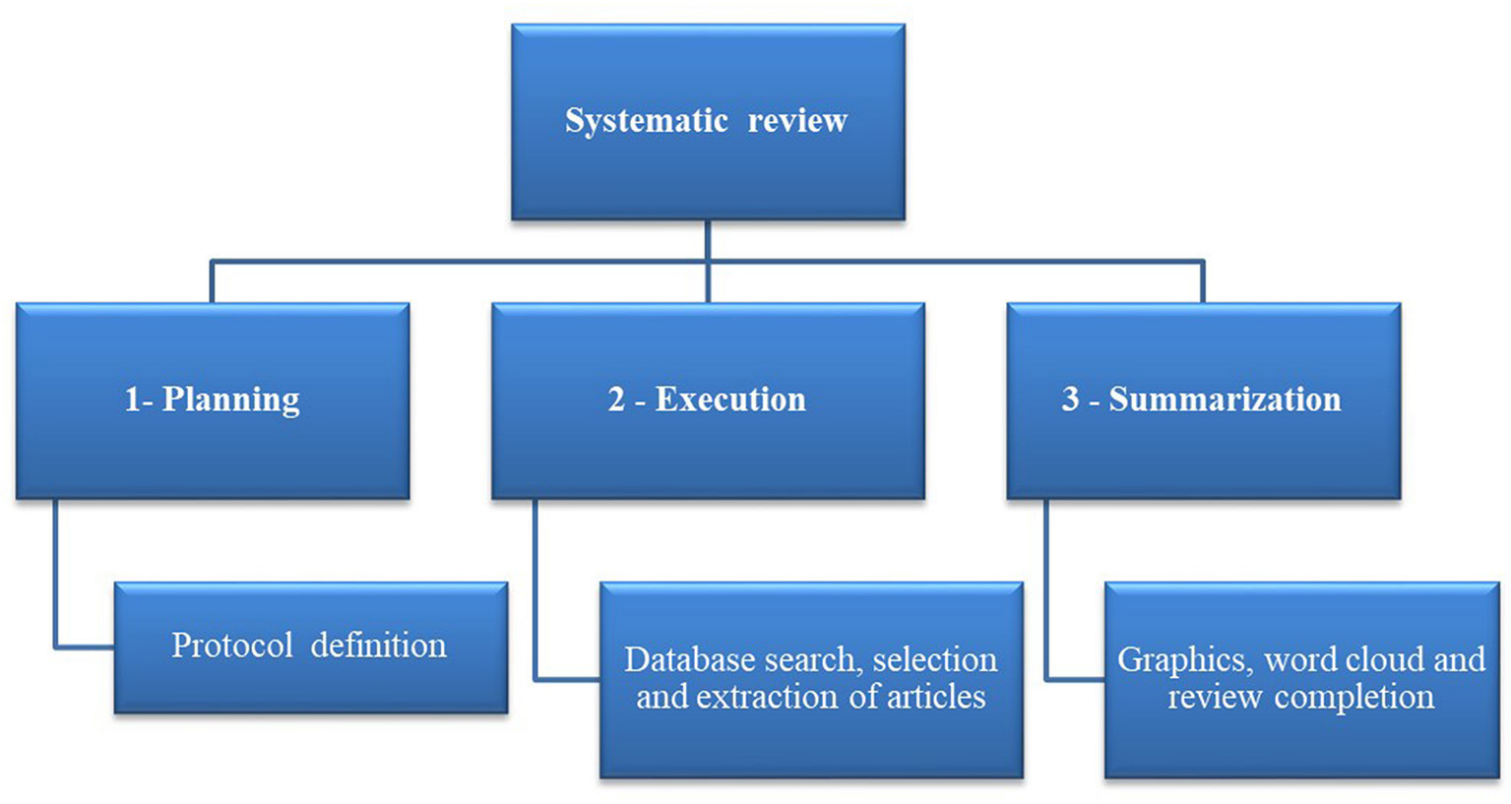
General systematic literature review flowchart [Adapted from Santos et al. (2018)].

The systematic review was conducted using the software StArt (State of the Art through Systematic Review) Beta version. 3.0.3, developed at the Federal University of São Carlos (UFSCar) to assist in systematic reviewing (Santos et al., 2018). The software is freely available at http://lapes.dc.ufscar.br/tools/start_tool. This review consisted of three fundamental steps, summarized in Figure 1.

### Planning

In this step, a defined protocol was followed according to the following information: article title, authors, objective, keywords, research questions, research sources, inclusion/exclusion criteria and definition of study type (https://doi.org/10.5281/zenodo.4437073). The questions raised in this review are listed in Table 1.

**Table 1:** List of questions raised in the review.

**Questions**
1. Which countries carried out most studies on the genetic improvement of banana related to black Sigatoka?
2. Which institutions/bodies work with this theme?
3. Which are the most studied *Musa* genotypes and varieties?
4. In terms of commercial cultivars, which are resistant and which are susceptible to *P. fijiensis*?
5. What types of trials are proposed in the studies?
6. Which genes are reported to be associated with resistance to black Sigatoka?
7. What are the biotechnological techniques employed in the studies?
8. What are the structural, genetic and molecular mechanisms involved in *Musa* defense responses responsible for conferring resistance to black Sigatoka?

### Execution

In order to answer the question of our research, “which strategies were adopted in genetic breeding to reduce the impact of black Sigatoka in bananas?” a research strategy of Population Intervention Comparison Outcome (PICOS), was used (de Costa Santos et al., 2007). This strategy guides what the research question really needs to specify avoiding a less biased answer (Wright et al., 2007). For it's elaboration, these following questions should be answered:

P–What is the research problem or who are the individuals populations?

I–What will be done, or which treatment or intervention or exposure?

C–Will any action intervention alternative treatment, or in parallel, be carried out?

O–What is the expected result or outcome?

S–What is the type of study?

The PICOS strategy used in this systematic review is shown in Table 2.

**Table 2:** Definition of the PICO terms of strategy for the question in the research used in this research.

**Description**	**Abbreviation**	**Components of the question**
Population	P	Banana plants *(Musa* spp.) with black Sigatoka
Interest/intervention	I	Genetic breeding methods used to control the disease.
Comparison	C	Lack of breeding methods or any other method of management or control of the disease, which does not involve genetic breeding (cultural, chemical, biological or other methods of control and management of the disease).
Outcome	O	Resistance or tolerance to black Sigatoka (basal or complete resistance)
Type of study	S	Scientific articles with experimental studies.

Searches were conducted in selected databases: CAPES journals (https://www.periodicos.capes.gov.br), PubMed Central (https://www.ncbi.nlm.nih.gov/pmc), Google Scholar (https://scholar.google.com.br), Springer (https://link.springer.com), Web of Science (http://apps.isiknowledge.com) and Scopus (https://www.scopus.com). The selected files were imported in BIBITEX and MEDILINE format compatible with StArt. Automated searches were made from the themes located in titles, keywords and summaries. Additional articles of relevance that were not identified automatically were subsequently added manually. For all databases, the same search string was employed, with connectors such as “or” and “and” used to group synonymous keywords and the main topics. The String employed was as follows: *Musa* spp. and bananas or plantains and black Sigatoka or *Mycosphaerella fijiensis* or *Pseudocercospora fijiensis* and genetic resistance and markers and genes.

### Summarization

This step comprised the elaboration of graphs, tables and a word cloud to summarize the systematic review. All articles that were selected during the selection and extraction phase were based on the following inclusion criteria: articles that contained the search string terms in the title, abstract or keywords; and articles that answered the protocol questions (Table 1). Criteria for exclusion were as follows: theses, dissertations, manuals, reports, book chapters, review articles, articles published in annals of events and studies without any clear contribution.

During the selection stage, articles imported into the software StArt were classified as accepted, rejected, or excluded due to duplication. In the extraction phase, a second selection was made considering only the articles that were accepted in the initial selection stage. During this phase, it was possible to delete duplicates, accept articles or reject those that were not in accordance with the objectives of the work, based on reading the articles in full, as well as on the inclusion and exclusion criteria. A PRISMA (Preferred Reporting Items for Systematic Reviews and Meta-Analyses) checklist is presented for download at https://doi.org/10.5281/zenodo.4659141.

## Results

### Database Searches

Aiming to reduce bias risks we opted to insert only articles with scientific and statistical data and also those which really considered our main and secondary questions whose conclusions were reliable. Regarding the specific evaluation of risk and bias tools used in clinical studies that were not yet adapted for use in other areas of knowledge also related to meta-analysis, were used. A PRISMA checklist was also used which is used strategically in systematic reviews aiming transparency and quality in the elaboration and publication of this review. Therefore, we guarantee that there is no bias risks in our review since all the PRISMA parameters were followed accordingly, guaranteeing reproducibility and reliability.

**Figure 2:**
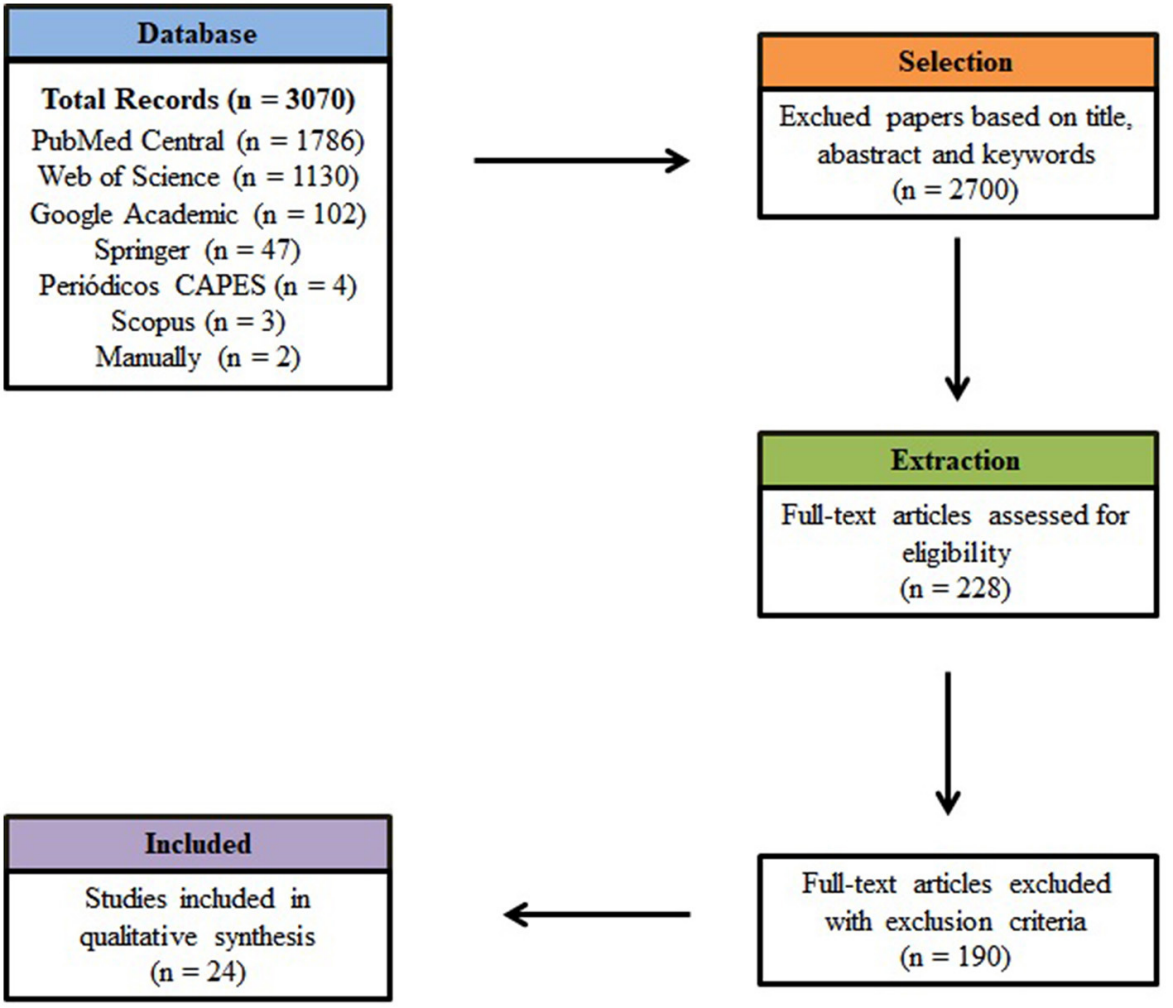
Prisma diagram for the screening process of articles selected in this review.

Electronic database searches using StArt resulted in the selection of 3,070 articles, published between January 2010 and December 2020. PubMed Central contributed with the largest number for this systematic review, corresponding to 1,786 papers, or 58% of the total. Web of Science contributed with 1,130 papers, representing 37% of those initially selected, followed by Google Academic (102), Springer (47), CAPES Journal (4) and Scopus (3). Although papers were selected using the search string, most were subsequently excluded from the study, as they were not related to the topic, and/or falling within the exclusion criteria. Two articles were also added manually (Figure 2).

During the initial evaluation of articles based on title and abstract, 2,070 articles did not meet the inclusion criteria. Together with 142 articles that were duplicated, these were all excluded from the systematic review. In the extraction stage, of the 228 remaining articles, 24 were accepted for analysis in the review from the criteria established for inclusion, as these answered the questions proposed in the initial protocol. For consultation purposes, these are stored in a free digital library at the following link: *https://doi.org/10.5281/zenodo*.

**Figure 3:**
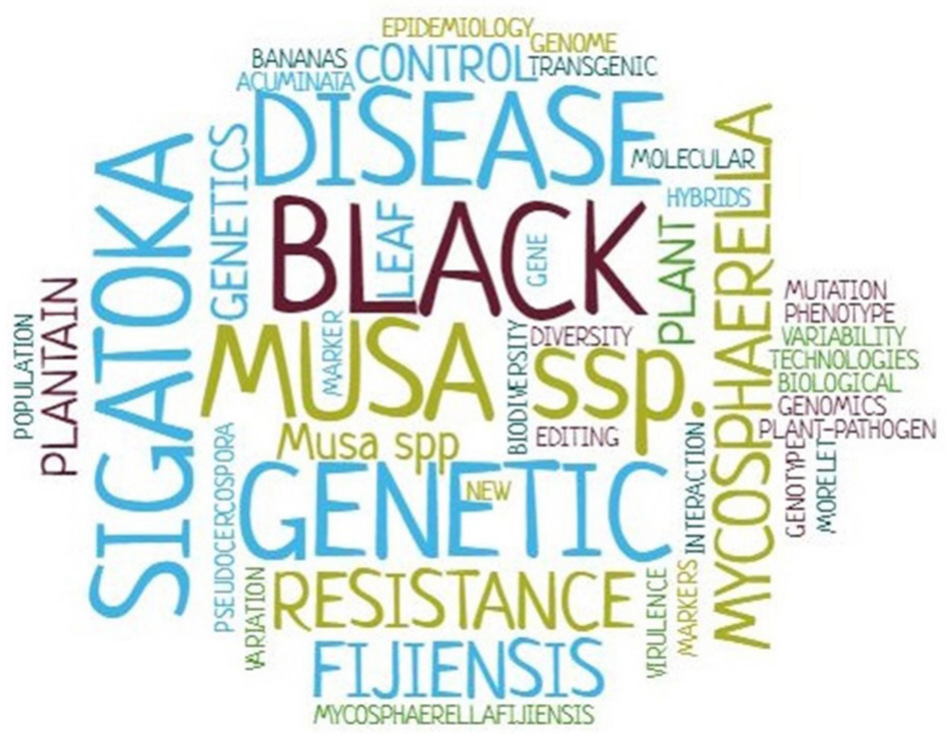
Word cloud based on the frequency of selected article keywords during the extraction phase of the systematic review into genetic improvement of banana for resistance to black Sigatoka.

A word cloud was generated during the extraction phase of the database search based on the frequency of keywords in the selected articles (*n* = 228). Highest frequencies of keywords in the articles were observed for black Sigatoka, *Mycosphaerella fijiensis, Musa* spp., disease and genetic resistance (Figure 3).

### Study Locations

**Figure 4:**
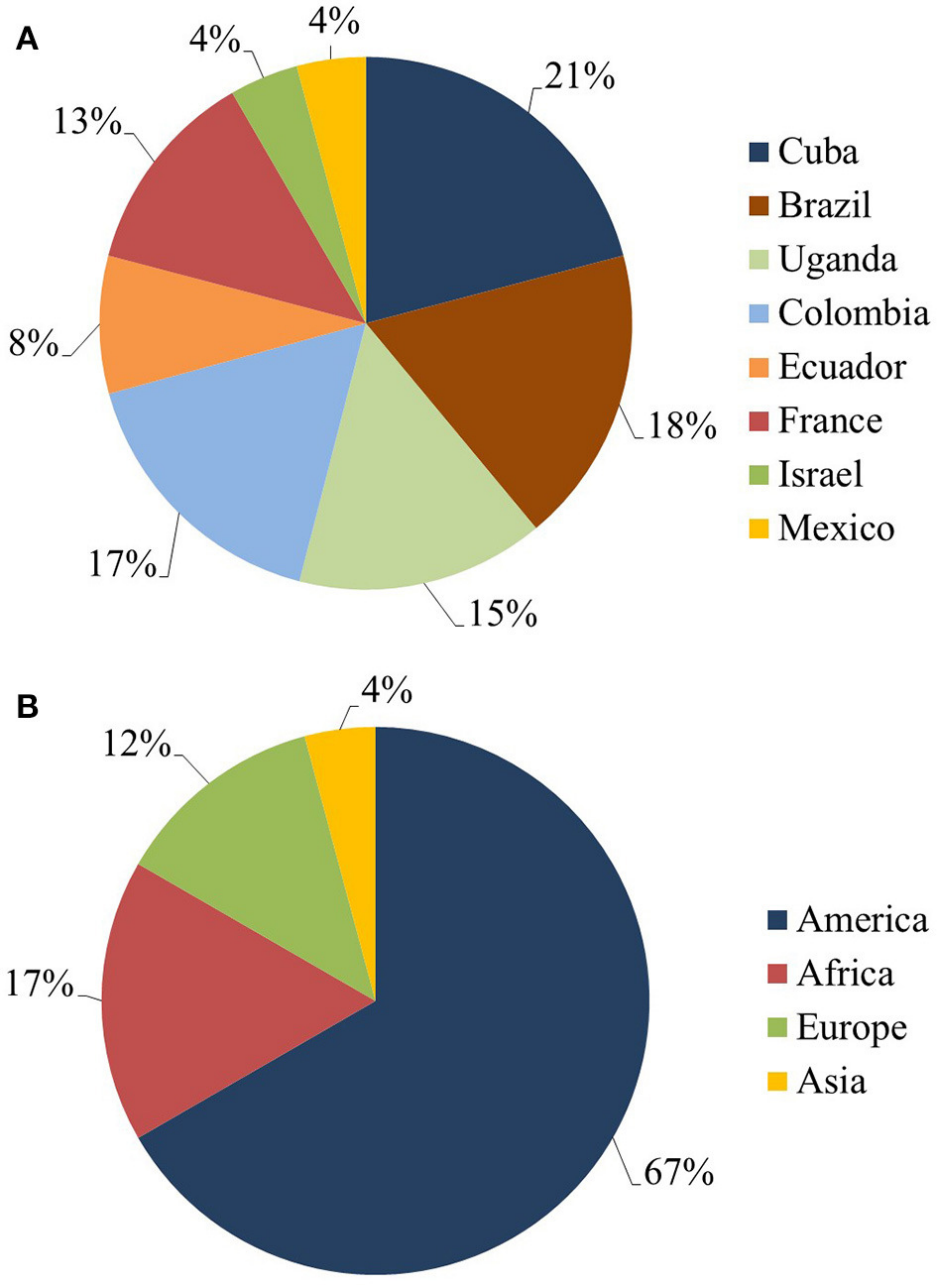
Summary pie charts for the published data from the last 10 years recognized in the systematic review into genetic improvement of banana for resistance to black Sigatoka. **(A)** Principal countries publishing data on resistance of *Musa* spp. to black Sigatoka. **(B)** Main continents to publish data on resistance of *Musa* spp. to black Sigatoka.

Most of the research work included in this systematic review was conducted in only three countries, namely Cuba (21%), Brazil (18%) and Colombia (17%). Belonging to the American continent, these represented the source of ~67% of the total 24 articles examined (Figure 4A). Articles from Africa, Europe and Asia represented 17, 13, and 4%, respectively (Figure 4B).

### Sources of Resistance and Study Environment

**Figure 5:**
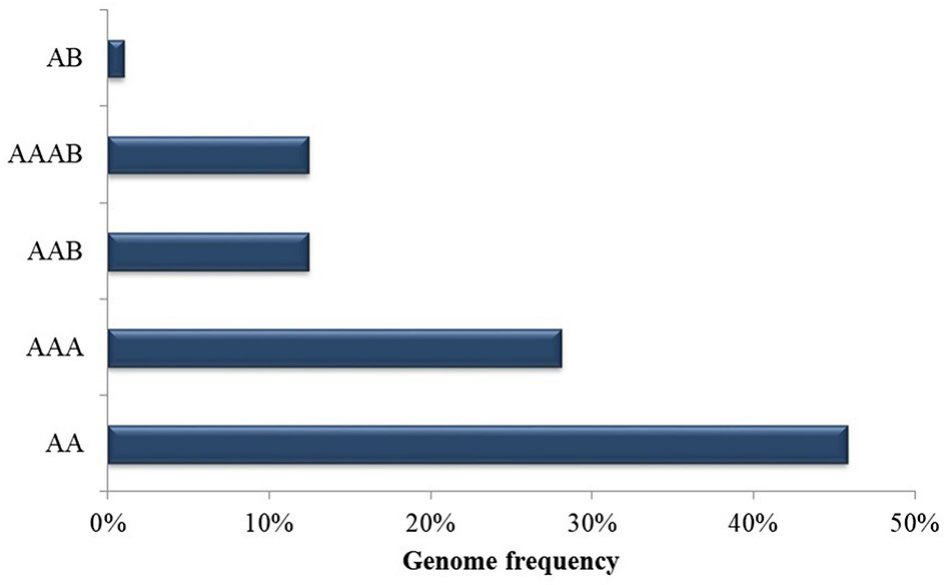
Genotype frequency of *Musa* spp. employed in published data recognized in the systematic review into genetic improvement of banana for resistance to black Sigatoka.

Cultivars and genotypes that are resistant, moderately resistant or susceptible to black Sigatoka were the object of study across the selected articles (Table 3). As summarized in Figure 5, most genotypes were diploid AA genome members, representing 46% of those studied, 28% were AAA triploids, 13% AAB genome triploids, 13% AAAB genome tetraploids, and 1% were AB genome diploids. Genotypes most widely employed in studies with *P. fijiensis* were identified as: *M. acuminata* Calcutta 4, Grande Naine and Williams. Although the majority of the resistant or moderately resistant genotypes were AA diploids, resistance was also reported across AAA, AAB, and AAAB members.

**Table 3:** *Musa* spp. genotypes most employed in published data recognized in the systematic review into genetic improvement of banana for resistance to black Sigatoka.

***Musa* genotype**	**Genomic group**	**Classification**
Calcutta 4	AA	Resistant
Orito	AA	Resistant
Birmanie	AA	Resistant
Krasan Saichon	AA	Resistant
Tuu Gia	AA	Resistant
Zebrina	AA	Resistant
N° 118	AA	Resistant
DH-Pahang	AA	Resistant
Pisang Lilin	AA	Moderately resistant
028003-01	AA	Moderately resistant
Buitenzorg	AA	Moderately resistant
Khi Maeo	AA	Moderately resistant
M53	AA	Moderately resistant
Malaccensis 1	AA	Moderately resistant
Malaccensis 2	AA	Moderately resistant
Malbut	AA	Moderately resistant
Mambee Thu	AA	Moderately resistant
Microcarpa	AA	Moderately resistant
Niyarma Yik	AA	Moderately resistant
PA Rayong	AA	Moderately resistant
Pisang Madu	AA	Moderately resistant
Pisang Cici	AA	Moderately resistant
Pisang Jaran	AA	Moderately resistant
Pisang Jari Buaya	AA	Moderately resistant
Pisang Lidi	AA	Moderately resistant
Pisang Pipit	AA	Moderately resistant
Pisang Rojo Uter	AA	Moderately resistant
Pisang Tongat	AA	Moderately resistant
SF-751	AA	Moderately resistant
Tjau Lagada	AA	Moderately resistant
Akondro Mainty	AA	Susceptible
Khai Nai On	AA	Susceptible
Pisang Berlin	AA	Susceptible
Tong Dok Mak	AA	Susceptible
IAC 1	AB	Susceptible
Yangambi Km5	AAA	Resistant
Kiwangaazi (M9)	AAA	Resistant
Grande naine	AAA	Susceptible
Williams	AAA	Susceptible
Filipino	AAA	Susceptible
Gross Michel	AAA	Susceptible
Guineo de seda	AAA	Susceptible
Guineo de Jardim	AAA	Susceptible
Guineo mulato	AAA	Susceptible
Guineo morado	AAA	Susceptible
Nakitembe	AAA	Susceptible
Limeño	AAB	Resistant
NAROBan1	AAB	Resistant
NAROBan2	AAB	Resistant
NAROBan3	AAB	Resistant
NAROBan4	AAB	Resistant
Thap Maeo	AAB	Resistant
Maqueño	AAB	Susceptible
Dominico	AAB	Susceptible
Dominico gigante	AAB	Susceptible
Dominico negro	AAB	Susceptible
Dominico-Hartón	AAB	Susceptible
Barraganete	AAB	Susceptible
PV42-68	AAAB	Resistant
Pacovan Ken	AAAB	Resistant
BRS Vitória	AAAB	Resistant
BRS Japira	AAAB	Resistant
BRS Preciosa	AAAB	Resistant
BRS Garantida	AAAB	Resistant
BRS Tropical	AAAB	Resistant
BRS Platina	AAAB	Resistant
BRS Maravilha	AAAB	Resistant
FHIA 02	AAAB	Resistant
FHIA 18	AAAB	Resistant

**Figure 6:**
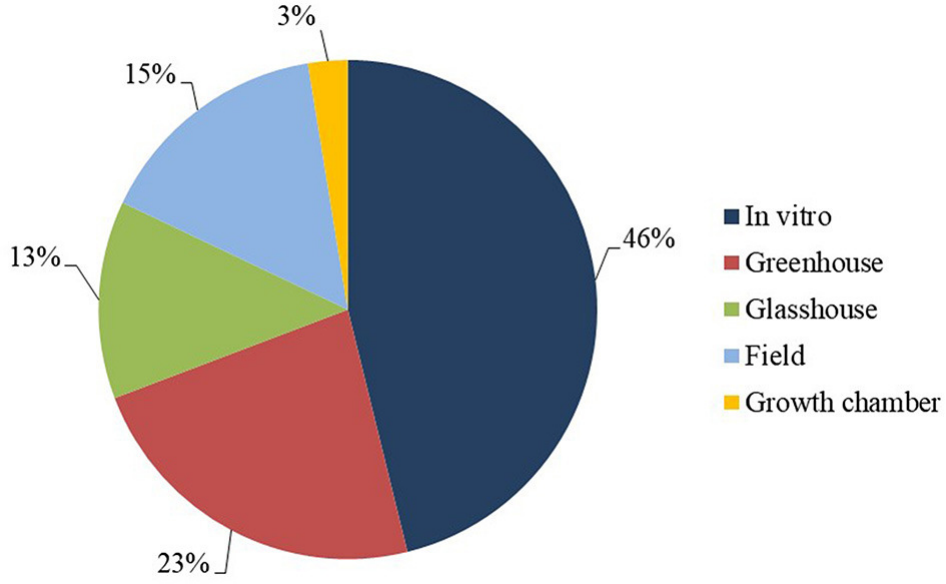
Study environment frequency.

With regard to the study environment, most of the studies were conducted on plant material *in vitro* (46%), followed by greenhouse (23%), field-based (15%) and glasshouse environments (13%) (Figure 6). *In vitro* work encompassed laboratory activities such as propagation of plants for testing, molecular analysis and fungal multiplication. Field work focused on analysis of agronomic characters, evaluation of resistance and other complementary analyses, such as consumer acceptance of resistant cultivars. In relation to greenhouse experiments, different pathogen inoculation approaches were utilized during evaluation of levels of resistance of different banana genotypes to *P. fijiensis* (Alvarado-Capó et al., 2003; Leiva-Mora et al., 2010).

### Methodologies Employed

**Figure 7:**
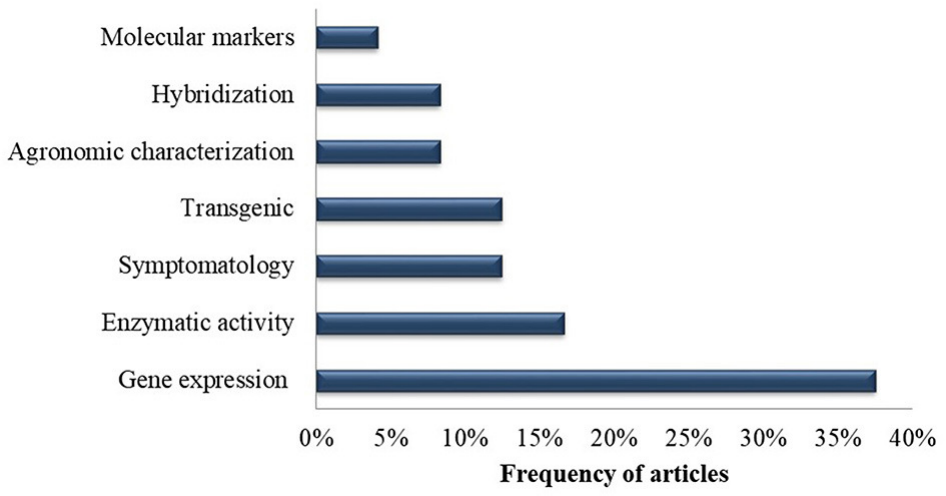
Frequency of methodologies utilized in the selected publications recognized in the systematic review in of genetic improvement of banana for resistance to black Sigatoka.

With regard to the main methodologies employed, gene expression analysis was addressed in 38% of the selected publications, followed by enzyme analysis (17%), symptomatology analysis (13%), transgenic development (13%), agronomic characterization (8%), *Musa* hybridization (8%) and characterization with molecular markers (4%) (Figure 7).

Leaf disease symptom evaluation employed grading scales that were proposed by Alvarado-Capó et al. (2003) and Stover (1972), as modified by Gauhl (1989). Three publications employing transgenic approaches were also identified in the study. Vishnevetsky et al. (2011) focused on the development of a transformation system for banana for pathogen control, with expression of the ThEn - 42 endochitinase gene from *Trichoderma harzianum*, together with a stybene synthase (StSy) gene resulting in transgenic events with improved tolerance to Sigatoka. Onyilo et al. (2018) conducted pathogen gene silencing approaches targeting mitogen-activated protein kinase pathogen genes Fus3 and Slt2, which are reported to be essential for pathogenicity. Portal et al. (2012) verified a green fluorescent protein-transformed *Mycosphaerella fijiensis* strain on susceptible banana “Grande Naine” and resistant “Yangambi km 5” plants, demonstrating that mutation events in *P. fijiensis* can increase virulence. In relation to agronomic characterization, two articles evaluated growth and production performance of genotypes resistant to black Sigatoka (Nowakunda et al., 2015; Weber et al., 2017). Enzymatic activity was also addressed in four publications that reported host enzyme actions during plant-pathogen interaction (Table 4). In two articles, *Musa* interspecific hybridization was also used to assess resistance development to black Sigatoka in progenies (Barekye et al., 2011; Tumuhimbise et al., 2018). Regarding molecular markers, one article addressed the development of microsatellite markers as a resource for *Musa* genetic improvement for resistance (Passos et al., 2012).

**Table 4:** Enzyme activities in *Musa* spp. during interaction with *Pseudocercospora fijiensis* in the selected publications recognized in the systematic review into genetic improvement of banana for resistance to black Sigatoka.

**References**	**Enzymatic activity in *musa* spp**.	**Function**
Cruz-Martín et al., 2018	APX - Ascorbate peroxidase	Antioxidant
	CHI – Chitinase ddharanii GLU - β-1, 3-glucanase	Degradation of invading pathogen cell wall polysaccharides
	PAL - phenylalanine ammonia lyase	Synthesis of plant defense compounds, such as phytoalexins
	POX – Phenol peroxidase	Synthesis of lignin
	SOD - Superoxide dismutase	Oxidative stress due to increased production of H_2_O_2_
Torres et al., 2012	CHI – Chitinase ddharanii GLU - β-1, 3-glucanase	Degradation of invading pathogen cell wall polysaccharides
	PAL - phenylalanine ammonia lyase	Synthesis of plant defense compounds, such as phytoalexins
	H_2_O_2_ – Peroxidase	Activates the plant's defense system
Mendoza-Rodríguez et al., 2017	H_2_O_2_ – Peroxidase	Activates the plant's defense system
Rodriguez et al., 2020	H_2_O_2_ – Peroxidase	Activates the plant's defense system

### *Musa* Gene Expression Analysis During Interaction With *P. fijiensis*

**Figure 8:**
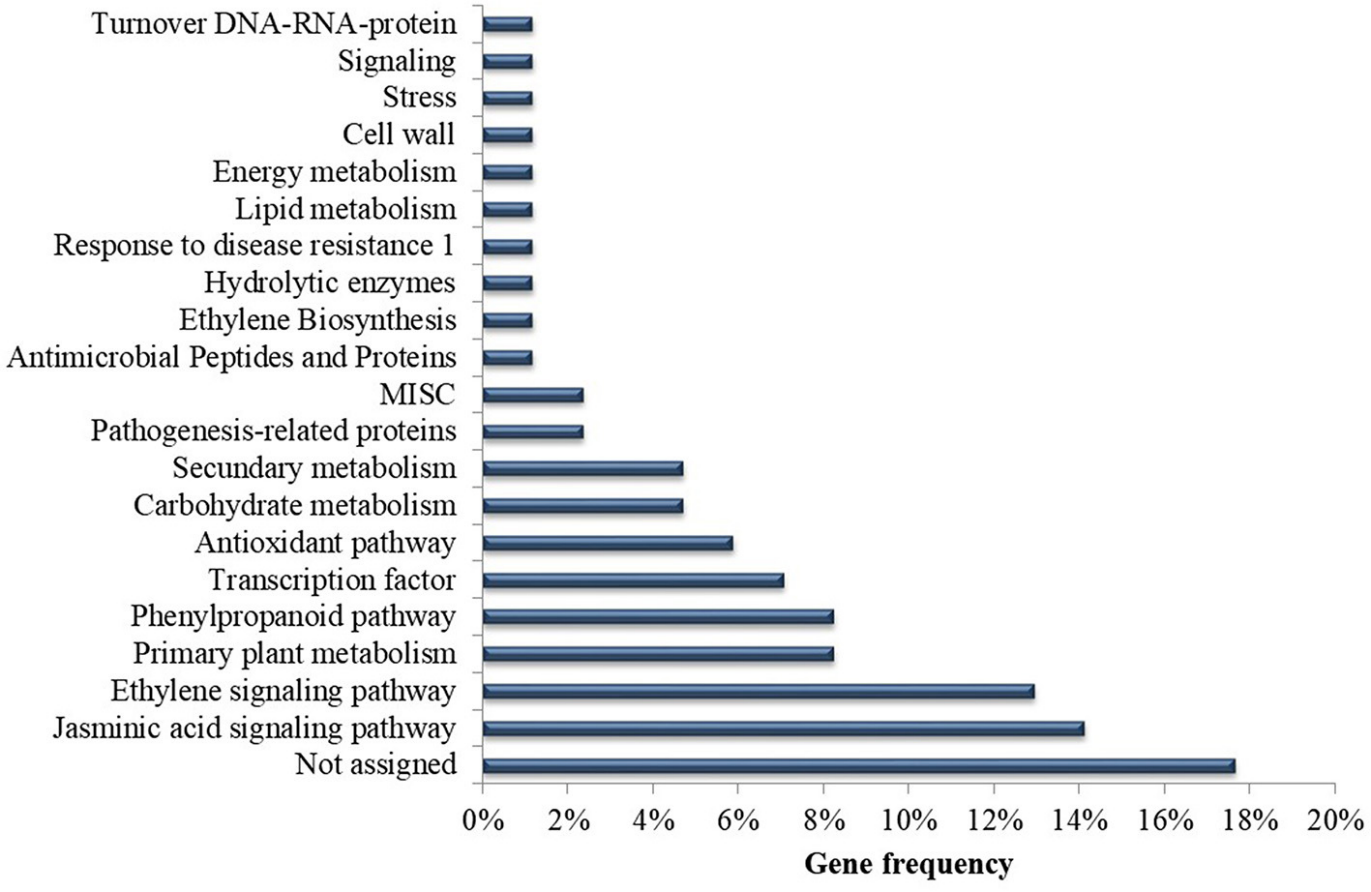
Frequency of analyzed *Musa* genes according to predicted function in the selected publications recognized in the systematic review of genetic improvement of banana for resistance to black Sigatoka.

Overall, eight articles (38%) investigated gene expression during the *Musa* x *P. fijiensis* interaction. Several candidate genes expressed differentially potentially involved in defense responses were identified in the selected articles (Supplementary Table 1). Of the genes identified in this systematic review, 18% are classified as an unassigned function, that is, the functions of these genes have yet to be discovered. The other genes are related to jasmonic acid signaling (14%), ethylene signaling (13%), primary metabolism (8%), secondary metabolism (8%), transcription factors (7%), via phenylpropanoid pathways (6%), antioxidants (6%), carbohydrate metabolism (5%), proteins related to pathogenesis (2%), among others (Figure 8) (Supplementary Table 1). In total, six different methods were used to inoculate the plants, with differences mainly in the form of application of spores on the leaf (brush or spray) and in relation to the concentration of spores, with values ranging from 1 × 10^3^ to 1 × 10^6^ (Supplementary Table 1).

### Enzymatic Activity

A total of 10% (*n* = 4) of the articles were related to analysis of enzyme activity in plants infected with *P. fijiensis* (Figure 7). In these publications, increased activity following inoculation was shown for the enzymes peroxidase (POX), phenylalanine ammonia lyase (PAL), β-1, 3-glucanase (GLU) and chitinase (CHI), superoxide dismutase (SOD), ascorbate peroxidase (APX), with elevated H_2_O_2_ production after infection and pathogen advance also shown (Table 4). In general, enzyme activity was investigated through comparison of resistant and susceptible genotypes after inoculation with *P. fijiensis*. One exception was the publication by Cruz-Martín et al. (2018), where enzymatic activity in *Musa* was analyzed in response to a strain of *Bacillus pumilus*.

## Discussion

### Database Searches

This review gathered articles published from January 2010 to December 2020 containing information related to studies on the genetic improvement of *Musa* spp. for resistance to *P. fijiensis*. Only articles that answered the questions established in the initial protocol were selected, with emphasis on genetic improvement of bananas and plantains. For this reason, first reports of the disease, articles on the genetic diversity of *P. fijiensis*, and strategies for disease management were not considered in the study. In addition, literature reviews were excluded in order to avoid underestimation of data, as data could theoretically be repeated when considering that the reviews published cite a large number of articles that are already present in our systematic review. In addition, we opted for articles that performed experimental analyses.

### Study Locations

Latin America accounts for 25% of the world's banana production and 80% of banana exports (FAOSTAT, 2021). Although Brazil is ranked fourth in terms of global banana production, production in the country is destined almost entirely to internal markets. Brazil and Cuba stand out in this study with the largest number of studies conducted within the objectives of this review. These countries, in addition to having adequate climates for the development of *P. fijiensis*, both employ irrigation systems for banana and plantain cultivation, potentially creating an environment favorable to the fungus. With the exception of certain high-altitude regions (> 1,500 m) (Costa Rica, Guatemala and Mexico), studies have shown that Central America has a natural rain scenario climate which is suitable for the persistence of *P. fijiensis*. In Latin America, Costa Rica is considered the second largest exporter of commercial bananas. Here, however, the black Sigatoka index is high, with fungicides applied up to 45 times a year in heavily infested areas (Yonow et al., 2019). In the main banana export cultivation areas of South America (Northern Colombia, Ecuador and Peru), the climate is less prone to the development of *P. fijiensis* when compared to Central America (Yonow et al., 2019). Here too, however, the number of fungicide cycles has increased considerably, particularly in Ecuador. This is likely due to reduced sensitivity of *P. fijiensis* populations to the widely employed fungicides (Jimenez et al., 2007). A study by Bebber (2019) on climate change related to black Sigatoka showed that in banana cultivation areas in Latin America and the Caribbean, the risk of infection has increased by a median of 44.2% since 1960. This is likely due to increased humidity and temperatures more favorable to the development of the pathogen. Although increased banana production and global trade have also probably facilitated the establishment and spread of black Sigatoka, climate change has made these regions more conducive to pathogen infection of plants (Bebber, 2019).

### *Musa* Breeding and Black Sigatoka Resistant Cultivars

The development of black Sigatoka resistant cultivars has been the focus of numerous breeding programs worldwide, with a number employing biotechnology as a support tool. The main banana breeding programs mentioned in the review are located in Africa, Asia and the Americas. In Africa, these comprise the International Institute of Tropical Agriculture (IITA), the National Research Organization (NARO), the Center Africain de Recherches sur Bananiers et Plantains (CARBAP) and the Center National de Recherche Agronomique (CNRA). In Asia, breeding programs are conducted at the National Banana Research Center (NRCB), the Indonesian Fruits Research Institute (ITFRI) and the Chinese Academy of Tropical Agricultural Sciences (CATAS). In the Americas, the Brazilian Agricultural Research Corporation (EMBRAPA), the Honduras Foundation for Agricultural Research (FHIA) and the Center de Coopération Internationale en Recherche Agronomique pour le Développement (CIRAD) are active in *Musa* improvement. These programs have made significant progress to date in breeding for resistance to black Sigatoka. The FHIA program developed a number of genotypes resistant to *P. fijiensis* that are now grown in different countries around the world, such as Uganda, Tanzania, Ghana, Kenya and Nigeria. In addition, these genotypes have also been employed in breeding programs at IITA, EMBRAPA, CIRAD, and CARBAP (Tenkouano and Swennen, 2004). IITA, together with NARO-Uganda, have also developed several East African cooking banana hybrids, known as NARITAs, which are high yielding and resistant to Black Sigatoka, with the most promising varieties now released to farmers (Ortiz, 2015). Hybrid plantains developed by IITA and considered resistant to *P. fijiensis*, known as PITAs, as well as resistant cooking hybrids, known as BITAs, are also available in countries such as Ghana, Ivory Coast, Cameroon, Uganda and Nigeria (Tenkouano and Swennen, 2004; Tenkouano et al., 2011). The EMBRAPA breeding program has also developed hybrid bananas that are currently being evaluated for resistance to *P. fijiensis* in Nigeria and Uganda by IITA, countries where bananas and plantains represent the principal food base (Amorim et al., 2021). Several hybrids developed by EMBRAPA also form the basis of banana production in Northern Brazil, a region widely affected by black Sigatoka. Cultivars included in an ongoing adoption process by Brazilian producers include BRS Platina, a Prata-type, together with BRS Princesa, a Silk-type hybrid, which are both resistant to black Sigatoka, and *Fusarium oxysporum* f. sp. *cubense* race 1. In addition to the above, CIRAD and CARBAP also have advanced breeding programs that have also developed banana hybrids resistant to *P. fijiensis* through conventional strategies. These programs employ colchicine to duplicate chromosomes and aim to develop new cultivars rather than improving available germplasm (Tomepke et al., 2004). CARBAP maintains the largest collection of *Musa* spp. in the world, with more than 700 varieties from various geographic regions, and more than 150 banana cultivars (group AAB) susceptible to black Sigatoka (Mourichon et al., 1997; Tomekpe et al., 2011). All of these breeding programs use crossing methods to obtain resistant materials.

### Sources of Resistance

Black Sigatoka seriously affects dessert banana cultivars such as those of the Cavendish subgroup. One of the possible reasons for this susceptibility may lie in the monoculture format adopted for this subgroup, which theoretically may provide a favorable environment both for the emergence of resistance to fungicides within the pathogen population, as well as individuals with different virulence and/or aggressiveness characteristics (Churchill, 2011). A second reason is due to the type of host response to the pathogen. Although a reaction of the plant to attack by the pathogen has been recognized, the magnitude and development over time is regarded as insufficient to stop the progress of the fungus (Churchill, 2011; Torres et al., 2012; Cruz-Martín et al., 2018). For these reasons, one of the main recommended alternatives to fungicide-based approaches for the control of black Sigatoka is through the replacement of susceptible cultivars, such as those within the Cavendish subgroup, with agronomically appropriate resistant cultivars (Churchill, 2011).

In the present study, cultivars were reported with different levels of resistance, being classified as resistant, moderately resistant or susceptible to black Sigatoka. Among the most widely employed genotypes in the selected publications, the resistant *M. acuminata* wild diploid Calcutta 4, widely employed in breeding programs, and the susceptible triploid Grande Naine (Cavendish), used in commercial plantations for export and local consumption, both stand out in terms of frequency. Wild diploid *M. acuminata* bananas possess AA genomes and can harbor important sources of resistance genes for the genetic improvement of triploid cultivars (Timm et al., 2016). Studies into gene expression in Calcutta 4 have served as an approach to reveal candidate genes for resistance to the disease and to elucidate the mechanisms of resistance involved in the hypersensitivity response (Arango-Isaza et al., 2016; Timm et al., 2016; Mendoza-Rodríguez et al., 2017). In addition to Calcutta 4, other diploids resistant and moderately resistant to *P. fijiensis* include: Krasan Saichon, Zebrina, Birmanie, No. 118, Tuu Gia, PA Rayong, Pisang Cici, Malaccensis 1, 028003-01, Microcarpa, Pisang Lidi, Pisang Lilin, and Malbut. These have served as parentals for generation of improved diploids for subsequent introgression of genes in new cultivars (Nascimento et al., 2020).

Among the cultivars resistant to *P. fijiensis* mentioned in this review, BRS Maravilha, BRS Platina, FHIA-02, FHIA-18, and Galil 18 have adequate size and high yield potential, and represent alternatives to the traditional Prata subgroup. BRS Princesa, BRS Tropical and Caipira have also been promoted as alternatives to Silk bananas, with the cultivar Buccaneiro also an alternative to susceptible cultivars of the Gros Michel subgroup and appropriate for irrigated agrosystems (Weber et al., 2017).

The cultivar BRS Preciosa can also replace the commercial varieties Prata and Pacovan, without jeopardizing acceptability (Garruti et al., 2012; Amorim et al., 2021). In our review, we did not identify options of resistance to black Sigatoka in any cultivars of the Cavendish subgroup. Banana genetic improvement programs have, however, been focused on this objective, with EMBRAPA, CIRAD and FHIA working on the development of pathogen resistant genotypes with similar fruit quality to Cavendish subgroup bananas.

### Host Immune Responses to *P. fijiensis*

The identification of physical and chemical barriers related to banana defense has been the object of study to understand the mechanism of resistance to *P. fijiensis*. Lignification, together with production of phytoanticipins, phenols, phenylphenalenones, peroxidases, PAL (phenylalanine ammonia lyase), β-1,3 glucanase, and hydrogen peroxide all increase during incompatible interactions (Hoss et al., 2000; Otálvaro et al., 2007; Cruz-Cruz et al., 2010; Cavalcante et al., 2011; Torres et al., 2012; Sanchez-García et al., 2013; Hidalgo et al., 2016; Alakonya et al., 2018).

The sequencing of the reference genome of the diploid species *Musa acuminata* DH Pahang is an important resource for *Musa* improvement and has advanced understanding of banana evolution. In this study, numerous genes were identified that encode proteins potentially related to conserved components of PTI and ETI in monocots (D' Hont et al., 2012).

Analysis of gene expression is important for the identification of genes involved in plant-pathogen interactions. The genes C4H (cinnamate-4-hydroxylase), CHS (chalcone synthase), IRL (isoflavone reductase) and PAL (phenylalanine ammonia) are all related to the phenylpropanoid pathway. In the selected studies in this systematic review, these genes displayed similar up-regulated expression profiles in infected Calcutta 4 in contrast to an absence of such expression modulation in the susceptible cultivars Grande Naine (Mendoza-Rodríguez et al., 2018) and Williams (Alvarez et al., 2013), which presupposes recognition of the pathogen in Calcutta 4 and the appropriate expression of defense responses. The regulation of genes related to phytohormone defense responses is not entirely resolved in *Musa* spp. (Portal et al., 2011), although signaling associated with jasmonic acid (JA), salicylic acid (AS) and ethylene (ET) also participate in defense responses against pathogens. A total of 24 genes in the selected articles were related to signal transduction regulated by plant hormones, such as JA and ET (Supplementary Table 1). All genes related to the JA signaling pathway were found to be overexpressed in Calcutta 4 after inoculation with *P. fijiensis* (Rodriguez et al., 2020), whereas in the susceptible cultivar Williams, the activation of JA and ET defense responses was marginal, slow or non-existent, indicating potential suppression by pathogen effectors (Rodriguez et al., 2020). Pathogenesis-related proteins (PR) are induced in host plants after pathogen infection. PR-4 has been shown to have antifungal activity, disrupting cell polarity and binding to chitin in the cell wall of the fungus (Bormann et al., 1999; Portal et al., 2011). PR-10 exhibits ribonuclease and antifungal activity against pathogens in *Arachis hypogaea, Jatropha curcas*, and *Crocus sativus* (Chadha and Das, 2006; Gómez-Gómez et al., 2011; Agarwal et al., 2013). Here, in Calcutta 4, increased expression of genes encoding pathogenesis-related proteins PR-4 and PR-10 were found during interaction with *P. fijiensis* (Portal et al., 2011; Rodriguez et al., 2016). In a study by Mendoza-Rodríguez et al. (2018), gene expression in the incompatible interaction in Calcutta 4 also reported positive regulation of the PSI gene (primary metabolism), TRX (an antioxidant) and SAMS (methyl cycle), suggesting roles in the defense response. In their work, negative regulation of genes from the phenylpropanoid pathway were also active in Grande Naine during initial phases of infection by *P. fijiensis*. Despite the advances in studies to date, further functional analyses of genes are warranted to validate use as candidate genes for resistance in susceptible banana cultivars (Timm et al., 2016). It is clear that there is no standardized protocol for studies of gene expression in banana during interaction with *P. fijensis*, which may be a contributing factor to differences in results obtained.

Enzymes related to the defense response to *P. fijiensis* have been identified at different time points during infection and colonization. Raised enzymatic activities have been reported to occur earlier in certain resistant genotypes than in susceptible cultivars. As an example, Calcutta 4 showed a rapid induction of several defense-related enzymes, with peroxidase (POX), phenylalanine ammonia lyase (PAL), β-1, 3-glucanase (GLU) and well as the production of hydrogen peroxide (H_2_O_2_) during the first 72 h after inoculation, when compared to cv. Williams (Torres et al., 2012). H_2_O_2_ has been postulated to perform multiple functions in plant defense, with this reactive oxygen species involved in the rapid defense response of the plant identified as a hypersensitivity response (HR) (Awwad et al., 2019). One study has reported the accumulation of H_2_O_2_ associated with hypersensitivity reactions in Calcutta 4, enabling the rapid response in containing the development of the pathogen (Cavalcante et al., 2011). The enzymes POD and SOD are closely associated to oxidative stress responses caused by an increase in H_2_O_2_. As such, increased activities in these enzymes, in addition to other antioxidant enzymes such as APX, have been described during incompatible responses (Cruz-Martín et al., 2018; Awwad et al., 2019). As the first enzyme in the phenylpropanoid pathway, the role of PAL in conversion of precursors in lignin biosynthesis has been well-elucidated. In relation to banana, however, its' role in the production of secondary metabolites such as phenylphenalenones and phytoanticipins, with potential activity against *P. fijiensis*, is poorly resolved (Hidalgo et al., 2009; Cruz-Cruz et al., 2010; Torres et al., 2012).

### Study Environments

In relation to study environment, *in vitro* studies were conducted in a considerable proportion of the selected articles (45%). These comprised laboratory experiments investigating gene expression, enzymatic activity analysis, and gene function validation through transgenic approaches. Greenhouse studies corresponded to 24% of the articles, with focus on bioassays for evaluation of gene expression in *Musa* leaf tissues following inoculation with *P. fijiensis*. Field studies, which corresponded to only 16% of the articles, mostly focused on agronomic characterization and acceptance of resistant cultivars, with the exception of Barekye et al. (2011), who evaluated the contribution of diploid and tetraploid genotypes to triploid progenies, and Nascimento et al. (2020), who phenotyped 31 diploid accessions of Embrapa's germplasm collection for resistance.

### Principal Techniques Employed

Evaluation of symptoms was described in 13% of the articles, with scales employed for measurement of black Sigatoka symptoms based on the quantification of percentage leaf area with characteristic lesions. In the selected articles, two different scales were cited: Fouré (1985), Alvarado-Capó et al. (2003) and Stover (1972), modified by Gauhl (1989). The main difference between the evaluation scales is that the former presents five evaluation stages for black Sigatoka in the greenhouse, whilst the latter describes six stages which can be used both in the greenhouse and in the field.

Amongst the techniques, one single study assessed surgical defoliation as a strategy for reducing disease severity (Jiménez and Brioso, 2018).

Transgenic approaches were also employed in 13% in the selected articles. Transformation protocols based on the use of fluorescent markers were employed with the pathogen to better understand the infection process in susceptible and resistant banana germplasm (Portal et al., 2012). Gene silencing strategies were also applied to determine gene function in the pathogen in relation to virulence (Onyilo et al., 2018). Vishnevetsky et al. (2011) developed a transformation system for improved tolerance to Sigatoka, with focus on endochitinase and stybene synthase candidate genes for resistance.

Hybridization and agronomic characterization represented only 9% of the frequency of the selected articles. The generation of banana triploids using this technique requires an understanding of the influence of the progenitors on potential resistance to black Sigatoka, as well as agronomic characteristics of the progenies generated (Barekye et al., 2011). Evaluation of growth and production of banana genotypes with resistance to *P. fijiensis* in comparison with cultivars susceptible to the disease was also carried out (Weber et al., 2017).

Among the biotechnological techniques employed, molecular markers such as gene-derived microsatellite markers have also been developed (Passos et al., 2012). These markers are appropriate for use in molecular genotyping and marker-assisted selection (MAS) in order to accelerate strategies for *Musa* genetic improvement.

## Limitations of the Review and Future Research

As this systematic review was highly specific to the *Musa* x *P. fijiensis* interaction with regard to genetic improvement for resistance, the number of studies was limited to only 24 articles suitable for inclusion. This indicates not only the need for further studies with this focus, but also that research trends may be focused more on other methods of controlling black Sigatoka in banana, such as those based on the use of fungicides or cultural control strategies for disease management.

Nevertheless, we strengthen as our closing remarks, that the banana genetic breeding for black Sigatoka based in the development of resistant cultivars through different methods is an efficient tool in the integrated management of the disease. It is possible, through genetic breeding to obtain basal, quatitative resistance, since complete resistance has not yet been reported for the Musa x *P. fijiensis* pathosystem due to its complexity, especially as to selection of resistance genes with higher effect, and this is common for most agricultural crops (Kushalappa et al., 2016; Pilet-Nayel et al., 2017; Nascimento et al., 2020). Therefore, decreasing the symptoms of black Sigatoka obtained with limitations in the development of the pathogen in the tissues combined with cultural practices that aim reduction of the inoculum in the cultivated area is the best strategy for mitigating the impacts of the disease.

Banana possesses numerous characteristics that make genetic improvement a laborious and complex task. Despite this, breeding programs maintain a sustainable global banana agribusiness through the development of cultivars resistant to the main diseases of the crop. The process is inevitably slow, as *Musa* is a long cycle species that requires years for precise agronomic analysis of a new genotype to be completed. Agronomic studies combined with genetic studies employing biotechnological tools do, however, provide essential information for continuous genetic improvement.

The information contained in the literature on genes involved in the interaction between *Musa* x *P. fijensis* is still relatively scarce, with the need for further focus on this pathosystem. Future advances in this direction will no doubt contribute to the elucidation of important processes occurring during this plant-pathogen interaction. In the short term, priorities for future studies are summarized below:

- In terms of accurate disease assessment, appropriate symptom scales are required that consider both greenhouse and field assessment, as symptomology can differ between these environments.
- Standardized inoculation protocols are recommended for the rapid screening of plants for resistance in greenhouse environments.
- Standardized protocols for analysis of gene expression in *Musa* during interaction with *P. fijiensis* are recommended, to reduce differences due to methodologies in results obtained by different research groups.
- The sources of resistance in *Musa* germplasm highlighted in the results are relevant for conventional breeding for development of disease resistant cultivars. No options for resistance to black Sigatoka were identified in any cultivars within the subgroup Cavendish.
- The development of a *Musa* x *P. fijiensis* interaction model at the molecular level is warranted, that infers how resistant genotype such as *M. acuminata* Calcutta 4 recognize the pathogen and develop a resistance response, as well as what types of weapons the pathogen launches to succeed in infection against susceptible genotypes.
- Gene editing based on CRISPR/Cas9 has been a recent major advance that can pave the way for large scale functional genomics, enabling validation and modification of candidate genes associated with characteristics such as resistance to biotic stresses (pathogens and pests) and tolerance to abiotic stresses (temperatures and extreme droughts). Although this approach has not yet been applied to the *Musa*-*P. fijiensis* pathosystem, it offers considerable potential for the development of banana varieties with multiple and durable resistance and tolerance (Tripathi et al., 2019, 2020).

## Conclusion

Invaluable tools and resources have been developed in recent years to further understand the interaction between *Musa* and *P. fijiensis*. These include reference genome sequences, bioinformatic tools, transcriptomic, proteomic, enzymatic, and histochemical data that have enabled identification of genes, proteins and intracellular events activated during pathogen invasion and host defense responses. Although breeding programs have developed hybrids resistant to *P. fijiensis*, the continued identification of additional sources of resistance is necessary, considering that resistance offered may have only a low durability, given the high variability of this fungus and potential appearance of aggressive pathogen variants.

The data collected in this systematic review highlight the considerable information accumulated in the last 10 years that is applicable for improvement of *Musa* for resistance to black Sigatoka. The *M. acuminata* genotype Calcutta 4 has been widely studied and can be a target for breeding programs and future studies. Certain questions can also be raised in relation to specific datasets highlighted here, such as which genes identified through expression studies as candidates for disease resistance are appropriate for transgenic or genetic editing systems, or which molecular markers are applicable in marker-assisted selection. The functional characterization of genes and proteins will advance understanding of function of these potential targets in the host, facilitating the development of novel disease control strategies.

## Data Availability Statement

The datasets presented in this study can be found in online repositories. The names of the repository/repositories and accession number(s) can be found in the article/Supplementary Material.

## Author Contributions

JS, AR, FN, and EA developed the initial protocol for the development of the review. AS assisted in the use of the software. JS, AR, and FN carried out the data processing and development of the methodology, results, and discussion. EA, CF, FH, VA, and RM provided technical guidance and research supervision. All authors collaborated in the writing - revision, editing process, read, and agreed with the published version of the manuscript.

## Conflict of Interest

The authors declare that the research was conducted in the absence of any commercial or financial relationships that could be construed as a potential conflict of interest.
