# Supplementary material for: Genetic Improvement for Resistance to Black Sigatoka in Bananas: A Systematic Review
Source: Front Plant Sci. 2021 Apr 21;12:657916. doi: 10.3389/fpls.2021.657916 (PMC8099173; doi:10.3389/fpls.2021.657916)
Supplement: Supplementary file 1 [file Table_1.DOCX]

Supplementary Material

# Supplementary Tables

**Supplementary Table 1.** Plant defense-related genes identified in *Musa* in the selected publications recognized in the systematic review into genetic improvement of banana for resistance to black Sigatoka.

| **Authors** | **Gene** | **Gene function** | **Pathogen inoculation method** |
| --- | --- | --- | --- |
| Portal et. al., 2011 | ACC OXIDASE | Ethylene biosynthesis | Fungal suspension (1x10^5^ mL^-1)^. Leaves inoculated using a brush (Alvarado-Capó et al., 2003) |
|  | C4H | Phenylpropanoid pathway |  |
|  | GDSL-LIKE LIPASE | Hydrolytic enzymes |  |
|  | JAR1 | Jasmine acid signaling pathway |  |
|  | OSMOTIN-LIKE | Antimicrobial protein |  |
|  | PR-4/PR-10 | Pathogenesis-related proteins |  |
| Vishnevetsky et al., 2011 | SOD | Antioxidant pathways | * |
| D´Hont et al., 2012 | Putative Ethylene-responsive transcription factor 1  Putative Ethylene-responsive transcription factor 1B  NAC domain-containing protein 71  Transcription factor MYB21  Putative Protein TIFY 5ª  Putative Protein TIFY 10B | Transcription factor | Fungal suspension (1x10^6^ mL^-1^). Leaves spray inoculated |
|  | Putative Whole genome shotgun sequence of line PN40024  Putative expressed protein  UPF0497 membrane protein 2  UPF0497 membrane protein 11  Putative Protein terminal ear1  Metallothionein-like protein 4A  Hypothetical protein  Expressed protein  RTE1-like | Not assigned |  |
|  | Probable alpha alpha-trehalose-phosphate synthase [UDP-forming] 9  Plant neutral invertase domain containing protein expressed  Putative Lysosomal beta glucosidase  Putative glucan endo-1 3-beta-glucosidase GVI (Fragment) | Carbohydrate metabolism |  |
|  | Membrane protein putative expressed  Putative Probable nitronate monooxygenase | MISC (Miscellaneous Compounds) |  |
|  | Phenylalanine ammonia-lyase 1  Caffeoyl-CoA O-methyltransferase | Phenylpropanoid biosynthesis |  |
|  | Putative Flavanone 3-dioxygenase  Putative Naringenin 2-oxoglutarate 3-dioxygenase | Flavonoid biosynthesis |  |
|  | Putative Cytochrome P450 71D10  Putative Cytochrome P450 71D7 | Secondary metabolism |  |
|  | Non-specific lipid-transfer protein | Lipid metabolism |  |
|  | Plasma membrane ATPase | Energy metabolism |  |
|  | Lichenase | Cell wall |  |
|  | Putative Probable glutathione S-transferase GSTU6 | Stress |  |
|  | Histidine-containing phosphotransfer protein 4 | Signaling |  |
|  | Putative DnaJ protein | Turnover DNA-RNA-protein |  |
| Alvarez et al., 2013 | PAL | Phenylpropanoid pathway | Fungal suspension (1x10^6^ mL^-1^). Leaves spray inoculated |
| Rodríguez et al., 2016 | PAL | Phenylpropanoid synthesis | Fungal suspension (1x10^6^ mL^-1^). Leaves spray inoculated (Alvarez et al., 2013). |
|  | POX | Antioxidant pathways |  |
|  | DRR1 | Response to disease resistance1 |  |
|  | PR4/PR10 | Pathogenesis-related proteins |  |
| Timm et al., 2016 | SSH1-E3  SSH1-C2  SSH1-D8  SSH1-G5 | Primary metabolism | Fungal suspension (2x10^4^ mL^-1^). Leaves spray inoculated (Jiménez et al., 2018). |
| Rodríguez-García et al., 2016 | MfAvr4 | Pathogen avirulence gene | Fungal suspension (1x10^3^ mL^-1^). Leaves inoculated using a brush. |
| Mendoza Rodriguéz et al., 2018 | PS I  PS II  SAMS | Primary metabolism | Fungal suspension (1x10^5^ mL^-1^). Leaves inoculated using a brush (Leiva-Mora et al., 2010). |
|  | CAT  TRX  APX | Via antioxidant |  |
|  | CHS  C4H  IRL | Phenylpropanoid route |  |
| Rodriguez et al., 2020 | LOX  AOS  AOC  OPR1  OPR2  COI  COI1  MYC2  MYC4  MYB  TIFY3B | Jasmine acid signaling pathway | Fungal suspension (1x10^6^ mL^-1^). Leaves spray inoculated (Alvarez et al., 2013). |
|  | EFR1  EFR1B  EFR4  EFR5  EFR9  EFR10  EFR11  EFR17  EFR105  EIN3  ZAT10 | Ethylene signaling pathway |  |

* ^No mention of inoculation method^
